# Clinical efficacy and putative analgesic mechanism of heat-sensitive moxibustion for chronic nonspecific low back pain: a single-arm before-after study based on the brain-gut-immune axis

**DOI:** 10.3389/fnint.2026.1894020

**Published:** 2026-08-25

**Authors:** Yinfei Xie, Cheng Li

**Affiliations:** Department of Acupuncture, Affiliated Hospital of Jiangnan University, Wuxi, Jiangsu, China

**Keywords:** brain-gut-immune axis, chronic nonspecific low back pain, heat-sensitive moxibustion, intestinal permeability, lipopolysaccharide, putative analgesic mechanism, single-arm study, β-endorphin

## Abstract

**Background:**

Chronic nonspecific low back pain (CNLBP) is one of the leading causes of disability worldwide. Growing evidence implicates brain-gut-immune axis dysregulation in the pathogenesis and perpetuation of chronic musculoskeletal pain. Heat-sensitive moxibustion (HSM), a refined form of traditional suspended moxibustion that targets heat-sensitized acupoints, has demonstrated preliminary analgesic efficacy, but its mechanisms through the brain-gut-immune axis remain unexplored.

**Objective:**

To describe the clinical changes following HSM in patients with CNLBP and to explore its putative analgesic mechanism in relation to modulation of the brain-gut-immune axis, as assessed by nine serum biomarkers spanning neuroendocrine (β-endorphin, cortisol), intestinal barrier (lipopolysaccharide, D-lactate), gut-brain serotonergic signaling (5-hydroxytryptamine), and immune-inflammatory (interleukin-6, tumor necrosis factor-α, interleukin-10, high-sensitivity C-reactive protein) dimensions.

**Methods:**

This single-arm, pre-post exploratory study enrolled 204 eligible CNLBP patients who received 20 sessions of HSM over four weeks. Primary outcomes were the Visual Analog Scale (VAS) and Oswestry Disability Index (ODI). Secondary outcomes included the Self-Rating Depression Scale (SDS), Self-Rating Anxiety Scale (SAS), and the nine-biomarker panel. Blood samples were collected at baseline and post-treatment. Paired t-tests or Wilcoxon signed-rank tests were used for pre-post comparisons. Treatment responders ( ≥ 50% VAS reduction) and non-responders were compared, and multivariable logistic regression was performed to identify predictors of treatment response.

**Results:**

Of 204 enrolled patients, 186 completed the study (attrition rate, 8.8%). VAS scores decreased significantly after treatment (5.87 ± 1.38 vs. 2.21 ± 1.32; *P* < 0.001; Cohen’s *d* = 2.71), as did ODI scores (33.86 ± 11.24 vs. 18.42 ± 9.36; *P* < 0.001; *d* = 1.49). SDS and SAS scores also improved significantly (*P* < 0.001). In the neuroendocrine dimension, β-endorphin increased and cortisol decreased (*P* < 0.001). In the intestinal barrier dimension, LPS and D-lactate were significantly reduced (*P* < 0.001). Serum 5-HT showed a trend toward elevation that did not reach significance after FDR correction (adjusted *P* = 0.094). In the immune-inflammatory dimension, IL-6, TNF-α, and hs-CRP decreased, while IL-10 increased (*P* < 0.001). A total of 117 patients (62.9%) were classified as treatment responders. Compared with non-responders, responders showed significantly greater modulation in 8 of 9 biomarkers (all FDR-adjusted *P* < 0.05; 5-HT excepted). Multivariable logistic regression identified baseline heat-sensitization intensity, baseline VAS, and baseline LPS as independent predictors of treatment response (area under the curve = 0.828). Exploratory mediation analysis suggested that β-endorphin, IL-6, hs-CRP, LPS, and cortisol changes partially mediated the association between treatment dose and pain improvement.

**Conclusion:**

Following HSM, CNLBP patients showed clinically meaningful pain relief and functional improvement. These uncontrolled observations are consistent with, but cannot confirm, a putative analgesic mechanism involving four-dimensional modulation of the brain-gut-immune axis: central endogenous opioid activation, intestinal barrier restoration, gut-brain serotonergic signaling regulation, and peripheral immune rebalancing. Baseline heat-sensitization intensity, pain severity, and intestinal permeability may serve as potential predictors of treatment response. As a single-arm study without a control group, these findings establish association and biological plausibility rather than causal efficacy, and warrant confirmation in a randomized controlled trial with a sham-moxibustion comparator.

## 1 Introduction

Chronic nonspecific low back pain (CNLBP) refers to low back pain that persists or recurs for over 12 weeks without an identifiable pathological cause, and it is among the most common musculoskeletal disorders worldwide. The Global Burden of Disease Study 2021 reported that low back pain has remained the leading cause of years lived with disability across age groups and regions, with a global point prevalence of about 7.5% and more than 600 million people affected (GBD 2021 Low Back Pain Collaborators, 2023). CNLBP accounts for roughly 85–90% of all low back pain cases. Its pathophysiology is complex and involves peripheral and central sensitization, psychosocial factors, and systemic inflammation. The disease burden is heavy, yet the mechanisms behind CNLBP are still poorly understood, and treatments that act on these mechanisms remain limited.

Pharmacological options for CNLBP, including nonsteroidal anti-inflammatory drugs, opioids, and muscle relaxants, offer only modest pain relief and often bring notable side effects and limited long-term benefit. The SPACE randomized controlled trial found that opioids were no better than non-opioid drugs at relieving pain over 12 months, while causing more adverse effects (Krebs et al., 2018). For this reason, major clinical guidelines, including those of the American College of Physicians (Qaseem et al., 2017) and the World Health Organization (World Health Organization, 2023), now place non-pharmacological therapies as the first-line option for CNLBP. Among these therapies, acupuncture and moxibustion have drawn increasing clinical interest, since they relieve pain with few side effects and can act on several physiological pathways at the same time.

Heat-sensitive moxibustion (HSM) is a modified form of traditional suspended moxibustion that developed from the theory of acupoint heat sensitization. Conventional moxibustion is applied at fixed acupoints, while HSM works in a different way. It locates and stimulates heat-sensitized points, where patients report non-local thermal sensations such as penetrating heat, spreading warmth, or transmission to distant regions, and these sensations are thought to mark an activated state of the acupoint. A multi-center, three-arm randomized controlled trial in 456 patients with lumbar disc herniation found that HSM at heat-sensitized points led to better outcomes than moxibustion at conventional acupoints or conventional drug therapy (Chen et al., 2014). This evidence concerns lumbar disc herniation rather than CNLBP, but it still supports the clinical value of targeting heat-sensitized points in lumbosacral pain. The neurobiological and immunological mechanisms behind the analgesic effect of HSM, however, have rarely been studied, and very little work has examined HSM through the brain-gut-immune axis.

The brain-gut-immune axis is a two-way communication network that connects the central nervous system, the enteric nervous system together with the intestinal barrier, and the immune system, through neural, endocrine, and immune pathways (Ho et al., 2025). A growing body of work suggests that this axis is also involved in chronic musculoskeletal pain, not only in classical visceral pain syndromes (Ratna et al., 2023). One recent study showed that transplanting fecal microbiota from fibromyalgia patients into germ-free mice produced mechanical allodynia, thermal hyperalgesia, and spontaneous pain behaviors, which is direct causal evidence that gut microbes can drive chronic pain outside the viscera, and an open-label clinical trial in the same study also showed that correcting gut dysbiosis reduced pain in fibromyalgia patients (Cai et al., 2025). Genetic epidemiological data point in the same direction. A two-sample Mendelian randomization study of 18,340 participants found possible causal links between certain gut microbial taxa, gut-derived metabolites such as serotonin, and the risk of low back pain (Su et al., 2023). A later Mendelian randomization study found several mediating pathways from gut microbiota to chronic pain that pass through brain structural connectivity, which is consistent with a gut-brain-pain axis (Ma, 2025). The concept of a gut-disc axis has also been proposed, in which gram-negative bacterial lipopolysaccharide (LPS) leaks from a damaged intestinal barrier into the systemic circulation, triggers low-grade inflammation, and may accelerate intervertebral disc degeneration (Morimoto et al., 2023). This provides a theoretical basis for measuring serum LPS in patients with spinal pain.

The gut microbiome is not the only link between the brain, gut, and immune system in chronic musculoskeletal pain. Long-term activation of the hypothalamic-pituitary-adrenal (HPA) axis during chronic pain raises cortisol levels, and elevated cortisol in turn disrupts intestinal tight junction proteins and increases paracellular permeability (De Punder and Pruimboom, 2015). When bacterial LPS leaks into the systemic circulation, a state termed metabolic endotoxemia, it binds Toll-like receptor 4 on innate immune cells. This drives pro-inflammatory cytokine release and sets up a vicious cycle in which intestinal barrier damage, systemic inflammation, and pain reinforce one another (Mohammad and Thiemermann, 2020). Separately, gut-derived serotonin (5-hydroxytryptamine, 5-HT), which accounts for approximately 90% of the body’s total serotonin production, has been reported to modulate both pain perception and mood through vagal afferent and serotonergic pathways, representing a distinct gut-brain neuromodulatory channel rather than a marker of intestinal permeability. Previous studies have also demonstrated that endogenous opioid peptides, particularly β-endorphin released from the hypothalamic arcuate nucleus, play a central role in descending pain inhibition and are involved in neuroimmune crosstalk. These converging lines of evidence suggest that the analgesic mechanism of interventions targeting the brain-gut-immune axis may operate simultaneously across neuroendocrine, intestinal barrier, gut-brain signaling, and immune-inflammatory pathways—yet no clinical study has evaluated these dimensions in an integrated manner in the context of moxibustion treatment for CNLBP. The present exploratory study therefore aimed to describe the changes in pain, disability, and psychological outcomes following a four-week HSM protocol in CNLBP patients, to explore the potential analgesic mechanism of HSM through simultaneous assessment of nine serum biomarkers spanning the neuroendocrine, intestinal barrier, gut-brain signaling, and immune-inflammatory dimensions of the brain-gut-immune axis, and to identify potential baseline predictors of treatment response for guiding personalized clinical application.

## 2 Materials and methods

### 2.1 Study design

This was a prospective, single-arm, before-after (pre-post) exploratory study conducted at the Acupuncture Department of the Affiliated Hospital of Jiangnan University, Wuxi, Jiangsu Province, China, from January 2024 to April 2025. Because the study compared outcomes before and after treatment within the same patients without a concurrent or untreated control group, it corresponds to a single-arm before-after (case-series) design; accordingly, it can characterize the changes that accompany HSM but cannot, on its own, establish a causal effect of HSM on the outcomes. The study protocol was reviewed and approved by the Institutional Ethics Committee of the Affiliated Hospital of Jiangnan University. The study was conducted in accordance with the Declaration of Helsinki, and all participants provided written informed consent prior to any study-related procedures. Reporting followed the Strengthening the Reporting of Observational Studies in Epidemiology (STROBE) guidelines.

### 2.2 Participants

Adults aged 18–65 years were eligible if they had a clinical diagnosis of CNLBP, taken to mean low back pain that had lasted more than 12 weeks and could not be attributed to a specific pathological cause such as fracture, infection, tumor, cauda equina syndrome, or radiculopathy. Three further conditions also had to be met. Participants needed a VAS pain intensity score of at least 3 at screening, must not have received acupuncture or moxibustion in the 4 weeks before enrollment, and any analgesic medication had to have been kept stable, or not used at all, for at least the 2 weeks before enrollment.

Participants were excluded if imaging or clinical examination had identified a specific cause for the low back pain, or if they had previously undergone lumbar surgery. Current intake of selective serotonin reuptake inhibitors (SSRIs), serotonin-norepinephrine reuptake inhibitors (SNRIs), or other serotonergic medications was also grounds for exclusion, as was a known diagnosis of inflammatory bowel disease, irritable bowel syndrome, or another gastrointestinal disorder that compromises intestinal barrier function. Other reasons for exclusion were the current use of systemic corticosteroids or immunosuppressants, pregnancy or lactation, an active psychiatric disorder needing pharmacological treatment, and severe hepatic, renal, or cardiovascular comorbidities.

### 2.3 Intervention

All patients underwent the same standardized HSM procedure; however, the specific acupoints treated were individualized for each patient according to the location of heat-sensitized points identified during scanning. Before each session, the practitioner used a lit moxa stick held about 3 cm above the skin to scan the lumbosacral region for heat-sensitized acupoints. Any point at which the patient described a characteristic heat-sensitization response was selected as a treatment target. The heat-sensitization response was defined as a non-local, propagated thermal sensation—namely penetrating heat, expanding warmth, or transmission of heat to sites remote from the stimulation point—that reflects a functionally activated state of the acupoint rather than nociceptive thermal hyperalgesia or allodynia (Chen et al., 2014; Liao et al., 2014). This response denotes an enhanced deqi-like propagated sensation evoked at a sub-noxious skin temperature and does not refer to the perception of the thermal stimulus as more painful or hotter than it physically is. When more than one heat-sensitized region was detected during scanning, the point evoking the strongest heat-sensitization response was designated as the dominant point, was treated, and its intensity was recorded as that session’s heat-sensitization intensity (HSI) score. The strength of the heat-sensitization response was graded on a four-point ordinal scale (0–3), where a score of 0 indicated no sensitization, 1 indicated mild local warmth only, 2 indicated moderate penetrating or expanding heat, and 3 indicated strong distant transmission. The score was recorded at every session, and the mean HSI score for each patient was calculated as the average of the dominant-point scores across the 20 sessions. In each session, the single most strongly heat-sensitized point was treated; suspended moxibustion was applied to this dominant point for 30–45 min per session and was continued until the heat-sensitization response had faded, that is, until the patient reported that the heat sensation had returned to localized warming only. Thus, one dominant heat-sensitized point was treated per session, and the 30–45-min duration refers to that single session rather than to multiple concurrently treated points. Depending on the scanning result, the dominant point was most often located at Shenshu (BL23), Dachangshu (BL25), Weizhong (BL40), or an Ashi point identified by heat-sensitization testing. Patients received five sessions per week for four consecutive weeks, for a total of 20 sessions. The treatment schedule of five sessions per week followed the intervention intensity used in the standardized clinical protocol for HSM and in the multicenter randomized controlled trial of HSM for lumbar disorders (Chen et al., 2014), a high session frequency being adopted to deliver sufficient cumulative thermal stimulation while the acupoints remained in the heat-sensitized state. All treatments were given by licensed acupuncturists who had at least 5 years of clinical experience with HSM and who had completed standardized training.

### 2.4 Outcome Measures

#### 2.4.1 Primary outcomes

Two outcomes served as primary endpoints, namely the VAS for pain intensity and the Oswestry Disability Index (ODI) for functional disability. The VAS used a 10-cm horizontal line, with 0 marking no pain and 10 the worst imaginable pain. The ODI contains 10 items that capture daily functional limitations, and total scores run from 0 to 100, with higher scores reflecting greater disability.

#### 2.4.2 Secondary outcomes

Secondary clinical outcomes were the Zung Self-Rating Depression Scale (SDS) and the Zung Self-Rating Anxiety Scale (SAS). A patient was classified as a treatment responder when VAS dropped by 50% or more from baseline to post-treatment, in line with the Initiative on Methods, Measurement, and Pain Assessment in Clinical Trials (IMMPACT) recommendations for substantial clinical improvement.

### 2.5 Serum biomarker panel

#### 2.5.1 Neuroendocrine dimension

β-endorphin (β-EP) is the main endogenous opioid peptide involved in descending pain inhibition, and it was measured by enzyme-linked immunosorbent assay (ELISA; CUSABIO Biotech, Wuhan, China; Cat. No. CSB-E06827h), with an intra-assay coefficient of variation (CV) below 8%. Cortisol is the principal output hormone of the HPA axis and connects stress, pain, and immune regulation. It was measured by ELISA (Nanjing Jiancheng Bioengineering Institute, Nanjing, China; Cat. No. H094-1-2), with an intra-assay CV below 6%.

#### 2.5.2 Intestinal barrier dimension

LPS is a bacterial endotoxin that is commonly used as a serum surrogate marker for intestinal permeability (Seethaler et al., 2021), and it was quantified by the Limulus Amebocyte Lysate (LAL) chromogenic endpoint assay (Xiamen Bioendo Technology Co., Ltd., Xiamen, China; Cat. No. EC80545S). LAL-based LPS quantification in serum is known to vary because of serum protein interference (Harm et al., 2024). To reduce this problem, all samples were handled with endotoxin-free consumables, heat-inactivated at 70 °C for 10 min before assay, and run in duplicate. Spike-and-recovery validation was also carried out on a subset of 40 samples, with a mean recovery rate of 85.6 ± 11.8%. D-lactate is a product of bacterial fermentation and reflects mucosal damage (Wang et al., 2025), and it was measured by a colorimetric enzymatic assay (Elabscience Biotechnology Co., Ltd., Wuhan, China; Cat. No. E-BC-K002-M).

### 2.5.3 Gut-brain signaling dimension

Serum 5-HT was measured by competitive ELISA (Nanjing Jiancheng Bioengineering Institute, Nanjing, China; Cat. No. H104-1-1). Serum 5-HT mainly reflects platelet serotonin storage, together with the release of serotonin during clotting, rather than free circulating serotonin. The measurement was therefore used as a surrogate for gut-derived serotonergic tone, while recognizing that serotonin transporter (SERT) activity and platelet biology may act as confounders. Patients on SSRIs or SNRIs had already been excluded under the study criteria.

#### 2.5.4 Immune-inflammatory dimension

Interleukin-6 (IL-6), tumor necrosis factor-α (TNF-α), and interleukin-10 (IL-10) were each measured by ELISA (Nanjing Jiancheng Bioengineering Institute, Nanjing, China; Cat. No. H007-1-2, H052-1-2, and H009-1-2, respectively), with intra-assay CVs below 10%. High-sensitivity C-reactive protein (hs-CRP) was measured by ELISA (Elabscience Biotechnology Co., Ltd., Wuhan, China; Cat. No. E-EL-H0043).

### 2.6 Sample size estimation

As this was an exploratory study without a control group, a formal power calculation for between-group comparison was not applicable. The target sample size was determined based on two considerations: first, the primary aim of estimating pre-post effect sizes with adequate precision, for which 180 completers would provide > 95% power to detect a medium effect size (Cohen’s *d* = 0.5) and > 80% power to detect a small-to-medium effect size (*d* = 0.3) at α = 0.05 for paired t-tests; second, the planned multivariable logistic regression with up to six candidate predictors, requiring a minimum of 60 events based on the recommendation of ≥ 10 events per variable. Assuming an attrition rate of approximately 10%, 204 participants were enrolled.

### 2.7 Statistical analysis

Continuous variables are presented as mean ± SD or median (IQR) according to distribution (Shapiro-Wilk test). Pre-post comparisons used paired *t*-tests or Wilcoxon signed-rank tests as appropriate, with effect sizes reported as Cohen’s *d* or *r* = Z/√N. The Benjamini-Hochberg procedure controlled the false discovery rate (FDR) at 5% across the nine biomarkers.

Associations between biomarker and clinical changes were assessed by Pearson or Spearman correlation. Responder versus non-responder change scores (Δ = post-treatment - baseline) were compared by Welch *t*-tests or Mann-Whitney U tests with Benjamini-Hochberg FDR correction. To explore whether the analgesic effect was mediated by the brain-gut-immune axis, single-mediator analyses were performed on standardized variables, with treatment dose (mean HSI score) as the independent variable, each biomarker change as the mediator, and pain improvement (−ΔVAS) as the outcome; indirect effects (a × b), their bias-corrected bootstrap 95% confidence intervals (5,000 resamples), and the proportion mediated (indirect/total effect) were estimated. Treatment response was additionally defined by a ≥ 30% VAS reduction as a sensitivity analysis. The 186 completers had no missing data on any analyzed variable, so complete-case analysis was used throughout. Predictors of response ( ≥ 50% VAS reduction) were screened by univariable logistic regression; variables with *P* < 0.20 entered a backward-stepwise multivariable model, whose performance was assessed by the AUC, the Hosmer-Lemeshow test, and bootstrap internal validation (1,000 resamples). Analyses used R 4.3.2 and SPSS 27.0, with two-tailed *P* < 0.05 considered significant.

## 3 Results

### 3.1 Participant flow

**FIGURE 1:**
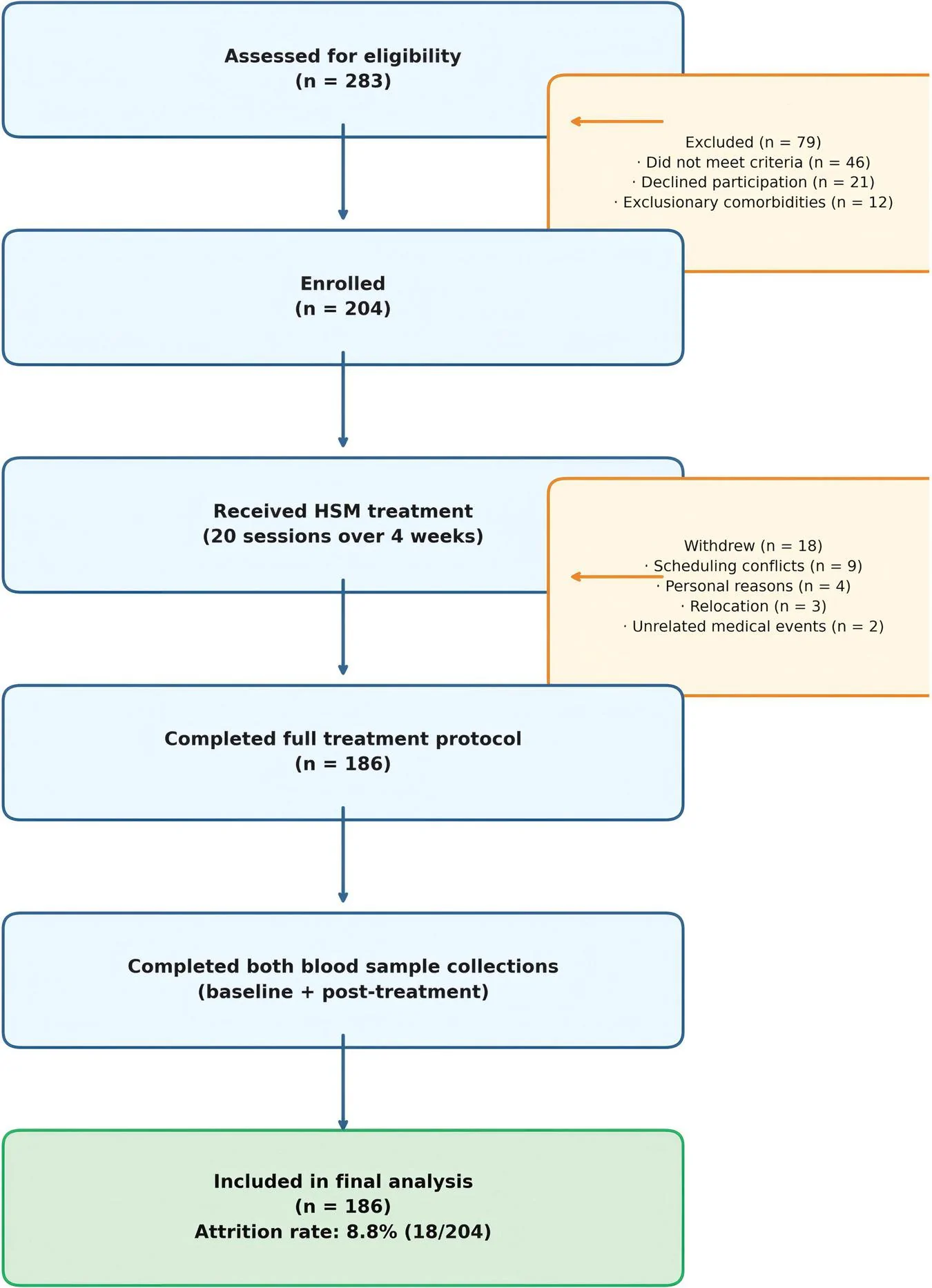
STROBE participant flow diagram. A total of 283 patients were screened; 79 were excluded (46 did not meet inclusion criteria, 21 declined participation, and 12 had exclusionary comorbidities); 204 were enrolled; 18 withdrew during treatment (9 scheduling conflicts, 4 personal reasons, 3 relocations, and 2 unrelated medical events); and 186 completed the full 20-session protocol and both blood sample collections, yielding an overall attrition rate of 8.8%. All analyses were based on the 186 completers.

Between January 2024 and March 2025, 283 patients were screened for eligibility. Of these, 79 were excluded (46 did not meet inclusion criteria, 21 declined participation, and 12 had exclusionary comorbidities), yielding 204 enrolled participants. During the treatment period, 18 patients withdrew: 9 due to scheduling conflicts, 4 for personal reasons, 3 due to relocation, and 2 due to unrelated medical events. Thus, 186 patients completed the full 20-session treatment protocol and both blood sample collections, yielding an overall attrition rate of 8.8% (18/204). All analyses were based on the 186 completers (Figure 1).

### 3.2 Baseline characteristics

The baseline demographic and clinical characteristics of the 186 completers are presented in Table 1. The mean age was 47.8 ± 11.5 years, with a female predominance (60.2%). The mean duration of low back pain was 36.8 ± 25.2 months. Baseline VAS was 5.87 ± 1.38, baseline ODI was 33.86 ± 11.24, and baseline SDS and SAS scores were 47.92 ± 9.48 and 45.73 ± 8.86, respectively. No significant differences were found between the 186 completers and the 18 dropouts in any baseline variable (all *P* > 0.10).

**TABLE 1:** Baseline demographic and clinical characteristics of participants (*N* = 186).

Characteristic	Value	Range/n (%)
Age (years)	47.8 ± 11.5	20–65
Sex (female), n (%)		112 (60.2%)
BMI (kg/m^2^)	23.9 ± 3.3	17.8–34.2
Pain duration (months)	36.8 ± 25.2	3–132
VAS	5.87 ± 1.38	3.0–9.4
ODI	33.86 ± 11.24	12–62
SDS	47.92 ± 9.48	25–74
SAS	45.73 ± 8.86	24–70
Current analgesic use, n (%)		52 (28.0%)
Smoker, n (%)		38 (20.4%)

Continuous variables are presented as mean ± SD; categorical variables as n (%). VAS, Visual Analog Scale; ODI, Oswestry Disability Index; SDS, Self-Rating Depression Scale; SAS, Self-Rating Anxiety Scale; BMI, body mass index.

### 3.3 Baseline serum biomarker levels

Baseline serum biomarker levels for the 9-biomarker panel are presented in Table 2, organized by the four sub-dimensions of the brain-gut-immune axis. All biomarker distributions were assessed for normality; LPS, D-lactate, IL-6, and TNF-α showed right-skewed distributions and are reported as median (IQR).

**TABLE 2:** Baseline serum biomarker levels by brain-gut-immune axis sub-dimension.

Sub-dimension	Biomarker	Mean ± SD or Median (IQR)	Unit	Distribution
Neuroendocrine	β-EP	154.6 ± 42.3	pg/mL	Normal
	Cortisol	18.52 ± 4.63	μg/dL	Normal
Gut barrier	LPS	27.8 (17.6–41.5)	EU/mL	Skewed
	D-lactate	16.2 (10.5–22.8)	μg/mL	Skewed
Gut-brain signaling	5-HT	121.3 ± 37.8	ng/mL	Normal
Immune-inflammatory	IL-6	8.56 (5.32–14.23)	pg/mL	Skewed
	TNF-α	12.18 (8.05–17.94)	pg/mL	Skewed
	IL-10	14.56 ± 5.72	pg/mL	Normal
	hs-CRP	4.72 ± 2.85	mg/L	Normal

β-EP, β-endorphin; LPS, lipopolysaccharide; 5-HT, 5-hydroxytryptamine; IL-6, interleukin-6; TNF-α, tumor necrosis factor-α; IL-10, interleukin-10; hs-CRP, high-sensitivity C-reactive protein. Normally distributed variables are presented as mean ± SD; skewed variables as median (interquartile range).

### 3.4 Pre-post changes in clinical outcomes

**FIGURE 2:**
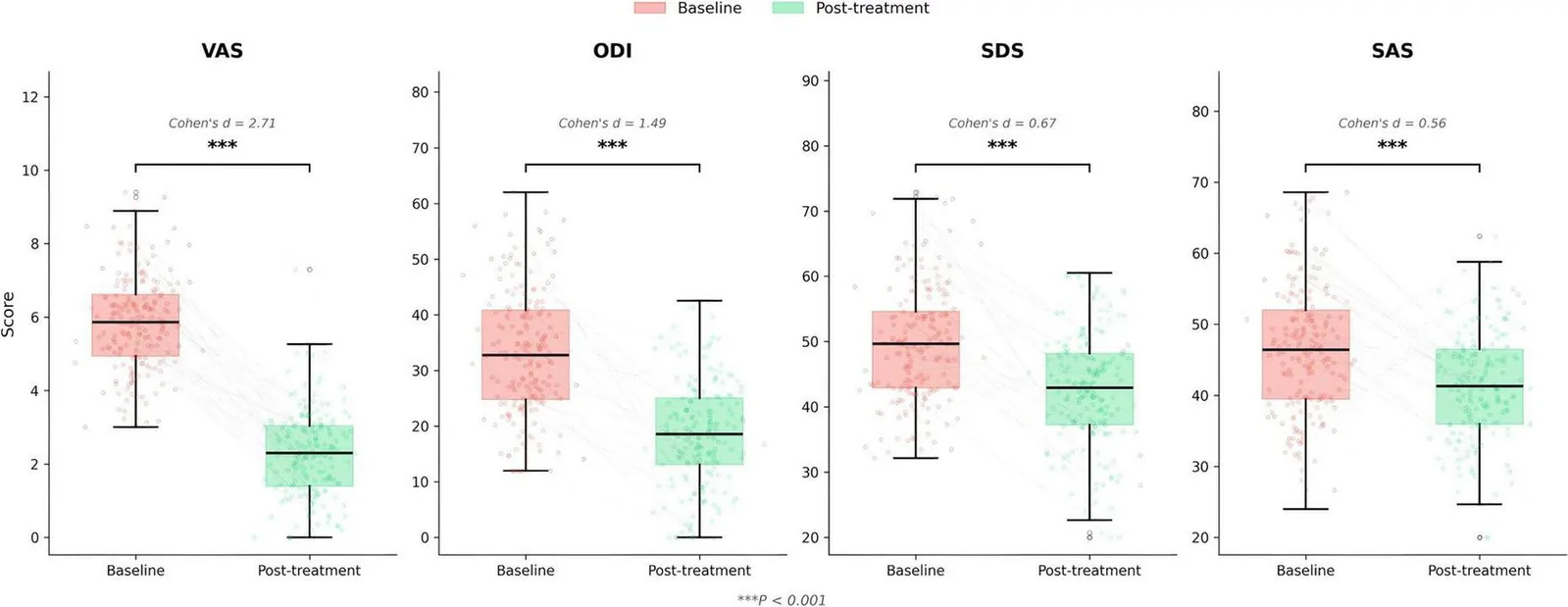
Pre-post changes in clinical outcome measures (VAS, ODI, SDS, SAS). Box plots with overlaid individual data points showing baseline and post-treatment distributions for each measure. The box represents the interquartile range, the horizontal line within the box indicates the median, and whiskers extend to 1.5 × IQR. ***P < 0.001 (paired *t*-test). VAS, Visual Analog Scale; ODI, Oswestry Disability Index; SDS, Self-Rating Depression Scale; SAS, Self-Rating Anxiety Scale.

All primary and secondary clinical outcome measures showed statistically significant improvement after the 4-week HSM treatment (Table 3). The VAS score decreased from 5.87 ± 1.38 to 2.21 ± 1.32 (mean change: −3.66, 95% CI: −3.95 to −3.37, *P* < 0.001), corresponding to a large effect size (Cohen’s *d* = 2.71). The ODI score decreased from 33.86 ± 11.24 to 18.42 ± 9.36 (mean change: −15.44, *P* < 0.001, *d* = 1.49). SDS and SAS scores also declined significantly (both *P* < 0.001), indicating concurrent improvement in psychological well-being. The mean HSI score remained stable across the 20 treatment sessions (Table 3; session 1, 2.10 ± 0.96 vs. session 20, 1.92 ± 1.05; *P* = 0.317 for the trend across all 20 sessions by Spearman correlation), indicating that the heat-sensitized state persisted throughout the treatment course rather than attenuating with repeated stimulation. Distributions of baseline and post-treatment scores are shown in Figure 2.

**TABLE 3:** Pre-post comparison of clinical outcome measures.

Outcome	Baseline	Post-treatment	Change (95% CI)	*t*/*Z*	*P*-value	Cohen’s *d*
VAS	5.87 ± 1.38	2.21 ± 1.32	−3.66 (−3.95, −3.37)	*t* = 25.84	< 0.001	2.71
ODI	33.86 ± 11.24	18.42 ± 9.36	−15.44 (−17.12, −13.76)	*t* = 18.28	< 0.001	1.49
SDS	47.92 ± 9.48	41.85 ± 8.72	−6.07 (−7.54, −4.60)	*t* = 8.17	< 0.001	0.67
SAS	45.73 ± 8.86	40.94 ± 8.25	−4.79 (−6.38, −3.20)	*t* = 5.95	< 0.001	0.56
Mean HSI score	2.10 ± 0.96	1.92 ± 1.05	−0.18 (−0.39, 0.03)	*t* = 1.68	0.095	0.12

VAS, Visual Analog Scale; ODI, Oswestry Disability Index; SDS, Self-Rating Depression Scale; SAS, Self-Rating Anxiety Scale; CI, confidence interval; HSI, heat-sensitization intensity. For the mean HSI score, “Baseline” corresponds to the session 1 score and “Post-treatment” to the session 20 score; the *P*-value is from the paired *t*-test comparing the session 1 and session 20 scores; the overall mean HSI across all 20 sessions was 2.06 ± 0.62.

### 3.5 Pre-post changes in serum biomarkers

**FIGURE 3:**
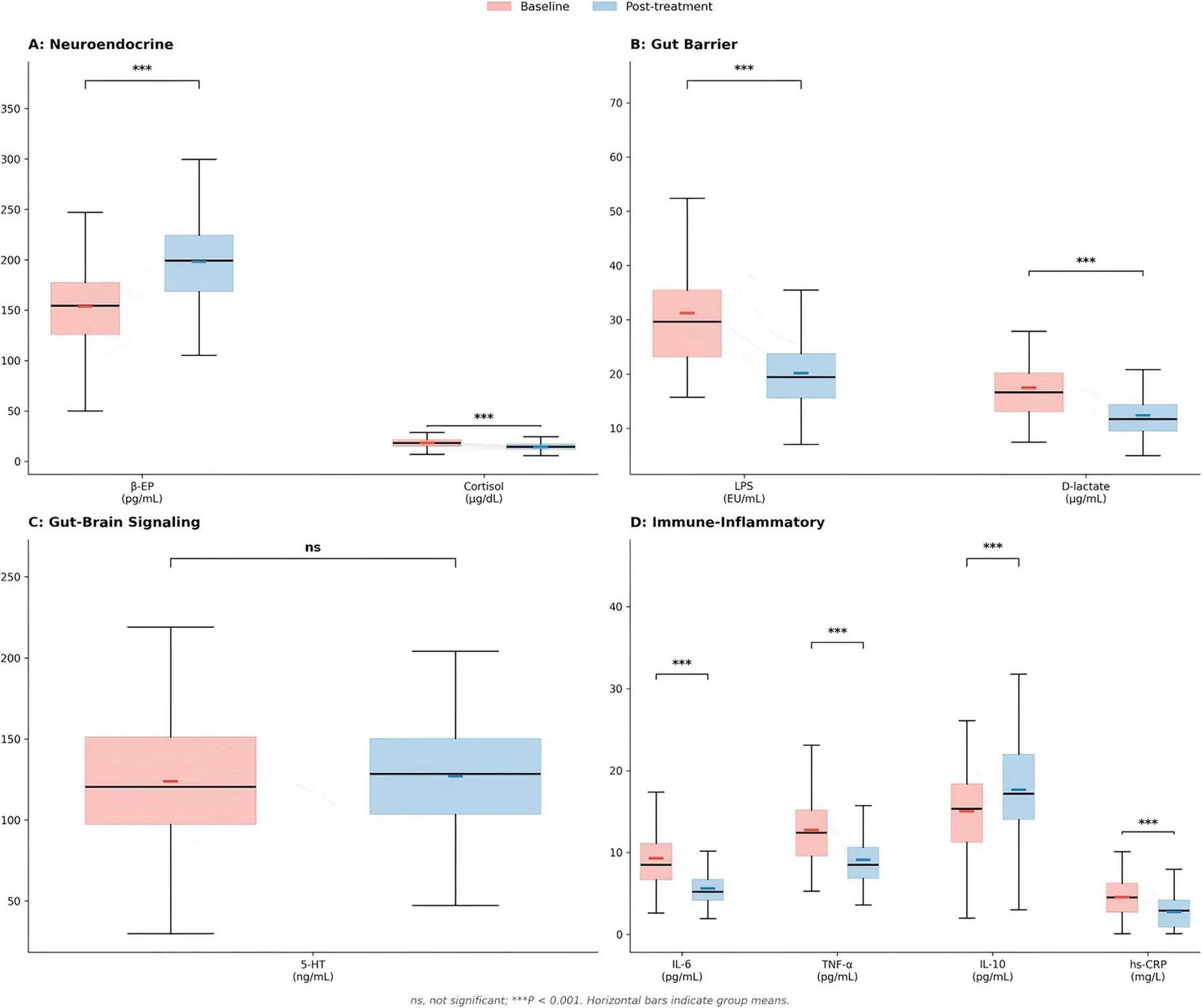
Pre-post changes in serum biomarkers by brain-gut-immune axis sub-dimension. **(A)** Neuroendocrine dimension (β-endorphin, cortisol). **(B)** Intestinal barrier dimension (lipopolysaccharide, D-lactate). **(C)** Gut-brain serotonergic signaling dimension (5-hydroxytryptamine). **(D)** Immune-inflammatory dimension (interleukin-6, tumor necrosis factor-α, interleukin-10, high-sensitivity C-reactive protein). Paired data are connected by lines; horizontal bars indicate group means. ns, not significant; ***P < 0.001 (Benjamini-Hochberg FDR-adjusted).

Table 4 presents the pre-post comparison of the 9 serum biomarkers organized by the four sub-dimensions. After Benjamini-Hochberg FDR correction, 8 of 9 biomarkers showed statistically significant changes. In the neuroendocrine dimension, β-EP significantly increased (*P* < 0.001), and cortisol significantly decreased (*P* < 0.001). In the intestinal barrier dimension, both LPS (*P* < 0.001) and D-lactate (*P* < 0.001) showed significant reductions. In the gut-brain signaling dimension, serum 5-HT demonstrated a numerical increase from 121.3 ± 37.8 to 125.8 ± 36.1 ng/mL, but this change did not reach statistical significance after FDR correction (unadjusted *P* = 0.063, FDR-adjusted *P* = 0.094). In the immune-inflammatory dimension, IL-6 (*P* < 0.001), TNF-α (*P* < 0.001), and hs-CRP (*P* < 0.001) showed significant decreases, while IL-10 significantly increased (*P* < 0.001). The pre-post distributions for each biomarker are shown in Figure 3.

**TABLE 4:** Pre-post comparison of serum biomarkers by brain-gut-immune axis sub-dimension.

Sub-dimension	Biomarker	Baseline	Post-treatment	*t*/*Z*	*P*-value	P-adj	Effect size
Neuroendocrine	β-EP (pg/mL)	154.6 ± 42.3	196.2 ± 44.8	*t* = -12.35	< 0.001	<0.001	*d* = 0.96
	Cortisol (μg/dL)	18.52 ± 4.63	14.38 ± 3.92	*t* = 11.84	< 0.001	<0.001	*d* = 0.97
Gut Barrier	LPS (EU/mL)	27.8 (17.6–41.5)	18.6 (11.8–27.4)	*Z* = -6.12	< 0.001	<0.001	*r* = 0.45
	D-lactate (μg/mL)	16.2 (10.5–22.8)	11.5 (7.8–17.6)	*Z* = -5.28	< 0.001	<0.001	*r* = 0.39
Gut-Brain	5-HT (ng/mL)	121.3 ± 37.8	125.8 ± 36.1	*t* = -1.87	0.063	0.094	*d* = 0.12
Immune	IL-6 (pg/mL)	8.56 (5.32–14.23)	5.28 (3.42–9.15)	*Z* = -7.42	< 0.001	<0.001	*r* = 0.54
	TNF-α (pg/mL)	12.18 (8.05–17.94)	8.52 (5.67–12.86)	*Z* = -6.18	< 0.001	<0.001	*r* = 0.45
	IL-10 (pg/mL)	14.56 ± 5.72	17.83 ± 6.28	*t* = -4.72	< 0.001	<0.001	*d* = 0.55
	hs-CRP (mg/L)	4.72 ± 2.85	2.53 ± 1.89	*t* = 10.62	< 0.001	<0.001	*d* = 0.91

β-EP, β-endorphin; LPS, lipopolysaccharide; 5-HT, 5-hydroxytryptamine; IL-6, interleukin-6; TNF-α, tumor necrosis factor-α; IL-10, interleukin-10; hs-CRP, high-sensitivity C-reactive protein; P-adj, Benjamini-Hochberg FDR-adjusted *P*-value.

### 3.6 Individual biomarker response patterns

Despite the overall significant trends, considerable inter-individual variability was observed in biomarker responses. For β-EP, 148 of 186 patients (79.6%) showed post-treatment increases, while 38 (20.4%) showed decreases or no change. For cortisol, 158 patients (84.9%) exhibited decreases. For LPS, 136 patients (73.1%) showed reductions, while 50 (26.9%) had stable or elevated post-treatment levels, possibly reflecting heterogeneity in baseline intestinal barrier status. For 5-HT, 108 patients (58.1%) showed increases, 64 (34.4%) showed decreases, and 14 (7.5%) had minimal change ( ± 5%), consistent with the non-significant group-level result and suggesting that serotonergic modulation by HSM may be subgroup-dependent.

### 3.7 Correlation analysis

**FIGURE 4:**
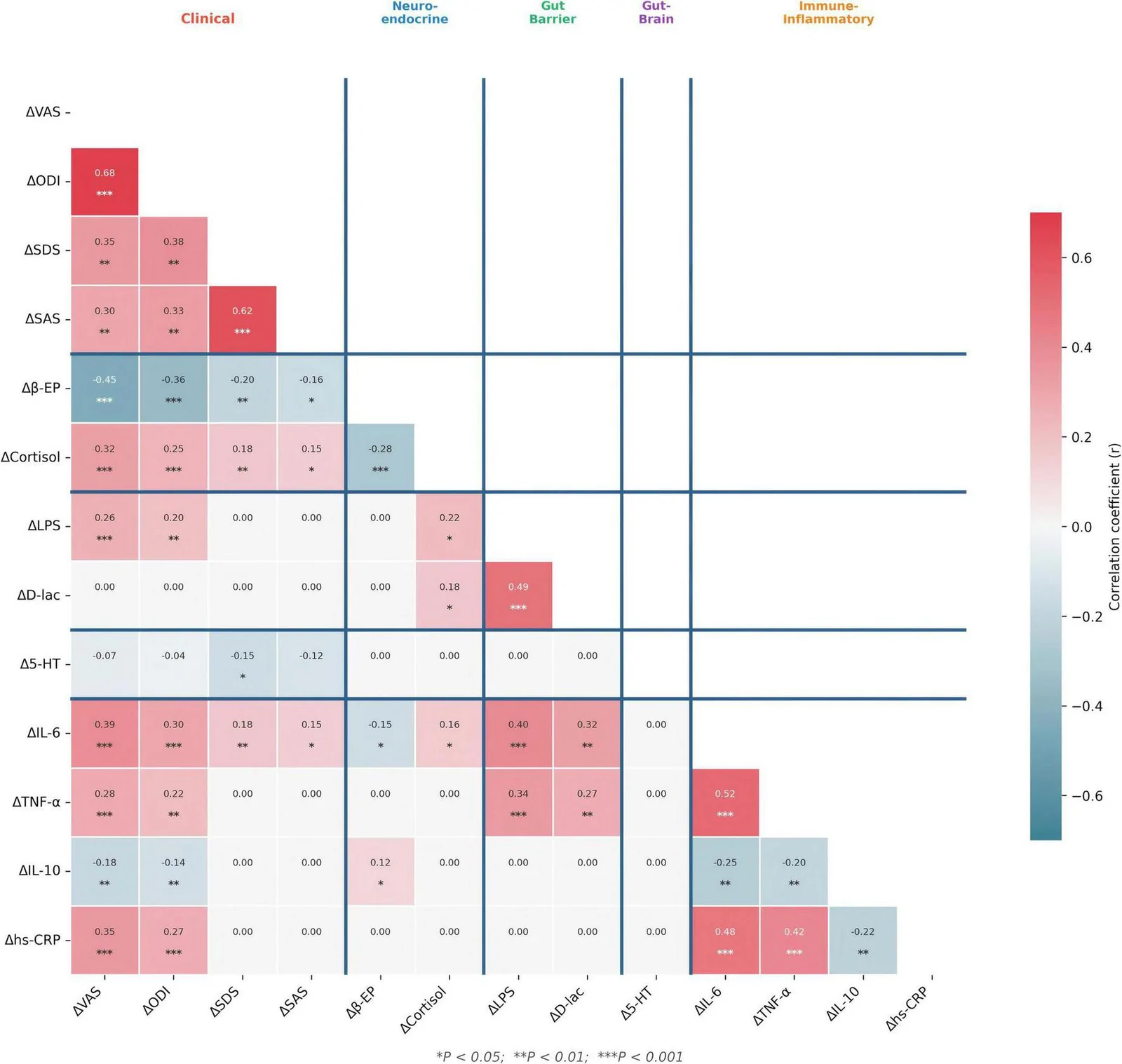
Correlation heatmap showing Spearman or Pearson correlation coefficients between changes (Δ) in serum biomarkers (Δβ-EP, ΔCortisol, ΔLPS, ΔD-lactate, Δ5-HT, ΔIL-6, ΔTNF-α, ΔIL-10, Δhs-CRP) and changes in clinical outcomes (ΔVAS, ΔODI, ΔSDS, ΔSAS). The color scale represents the correlation coefficient (r). Positive correlations are shown in warm colors and negative correlations in cool colors. Statistically significant correlations are indicated by asterisks (*P < 0.05; **P < 0.01; ***P < 0.001).

Correlation analysis between changes in biomarkers (Δ) and changes in clinical outcomes revealed several notable associations (Figure 4). Δβ-EP was negatively correlated with ΔVAS (*r* = -0.45, *P* < 0.001) and ΔODI (*r* = -0.36, *P* < 0.001), indicating that greater β-EP increases were associated with larger pain and disability reductions. ΔCortisol was positively correlated with ΔVAS (*r* = 0.32, *P* < 0.001). ΔLPS showed a significant positive correlation with ΔIL-6 (*r* = 0.40, *P* < 0.001) and ΔTNF-α (*r* = 0.34, *P* < 0.001), supporting the hypothesized pathway of intestinal barrier disruption driving systemic inflammation. ΔLPS also correlated significantly with ΔVAS (*r* = 0.26, *P* < 0.001). ΔD-lactate correlated with ΔLPS (*r* = 0.49, *P* < 0.001), confirming convergent validity of the two intestinal barrier markers.

Δ5-HT showed a weak but statistically significant negative correlation with ΔSDS (*r* = -0.15, *P* = 0.041) and a trend-level negative correlation with ΔSAS (*r* = -0.12, *P* = 0.098), suggesting a possible role for serotonergic modulation in mood improvement. However, no significant correlation was found between Δ5-HT and ΔVAS (*r* = -0.07, *P* = 0.34) or ΔODI (*r* = -0.04, *P* = 0.58), indicating that the modest serotonergic changes observed were more closely associated with the affective-motivational dimension of the pain experience than with sensory-discriminative pain intensity. Among immune markers, ΔIL-6 was significantly correlated with ΔVAS (*r* = 0.39, *P* < 0.001) and ΔODI (*r* = 0.30, *P* < 0.001).

### 3.8 Treatment responders versus non-responders

Based on the ≥ 50% VAS reduction criterion, 117 patients (62.9%) were classified as treatment responders and 69 (37.1%) as non-responders. Table 5 compares baseline characteristics between the two groups. Responders had significantly higher baseline VAS scores (6.28 ± 1.24 vs. 5.17 ± 1.31, *P* < 0.001), higher baseline LPS levels (32.6 [21.4–46.8] vs. 21.2 [13.8–31.7] EU/mL, *P* < 0.001), higher mean heat-sensitization intensity scores across the 20 sessions (2.35 ± 0.54 vs. 1.72 ± 0.58, *P* < 0.001), and higher baseline IL-6 levels (*P* = 0.005). Baseline ODI (*P* = 0.013) and hs-CRP (*P* = 0.011) were also significantly higher in responders. No significant differences were observed in baseline age, sex, BMI, pain duration, β-EP, cortisol, or 5-HT between the two groups.

**TABLE 5:** Baseline comparison between treatment responders and non-responders.

Variable	Responders (*n* = 117)	Non-responders (*n* = 69)	t/Z/χ ^2^	*P*-value
Age (years)	48.2 ± 10.9	47.1 ± 12.5	*t* = 0.63	0.531
Female, n (%)	71 (60.7%)	41 (59.4%)	χ^2^ = 0.03	0.862
BMI (kg/m^2^)	24.1 ± 3.2	23.5 ± 3.5	*t* = 1.22	0.224
Pain duration (months)	38.4 ± 26.8	34.1 ± 22.3	*t* = 1.14	0.257
Baseline VAS	6.28 ± 1.24	5.17 ± 1.31	*t* = 5.86	< 0.001
Baseline ODI	35.42 ± 10.86	31.22 ± 11.48	*t* = 2.52	0.013
Mean HSI score	2.35 ± 0.54	1.72 ± 0.58	*t* = 7.52	< 0.001
Baseline β-EP (pg/mL)	152.4 ± 41.8	158.3 ± 43.2	*t* = -0.92	0.358
Baseline Cortisol (μg/dL)	18.84 ± 4.72	17.98 ± 4.48	*t* = 1.22	0.224
Baseline LPS (EU/mL)	32.6 (21.4–46.8)	21.2 (13.8–31.7)	*Z* = -4.18	< 0.001
Baseline 5-HT (ng/mL)	122.8 ± 38.6	118.7 ± 36.4	*t* = 0.72	0.471
Baseline IL-6 (pg/mL)	9.52 (5.86–15.42)	6.94 (4.25–11.28)	*Z* = -2.84	0.005
Baseline hs-CRP (mg/L)	5.12 ± 3.04	4.05 ± 2.42	*t* = 2.58	0.011

Continuous variables as mean ± SD or median (IQR); categorical variables as n (%). HSI, heat-sensitization intensity. Treatment response defined as ≥ 50% VAS reduction from baseline.

**FIGURE 5:**
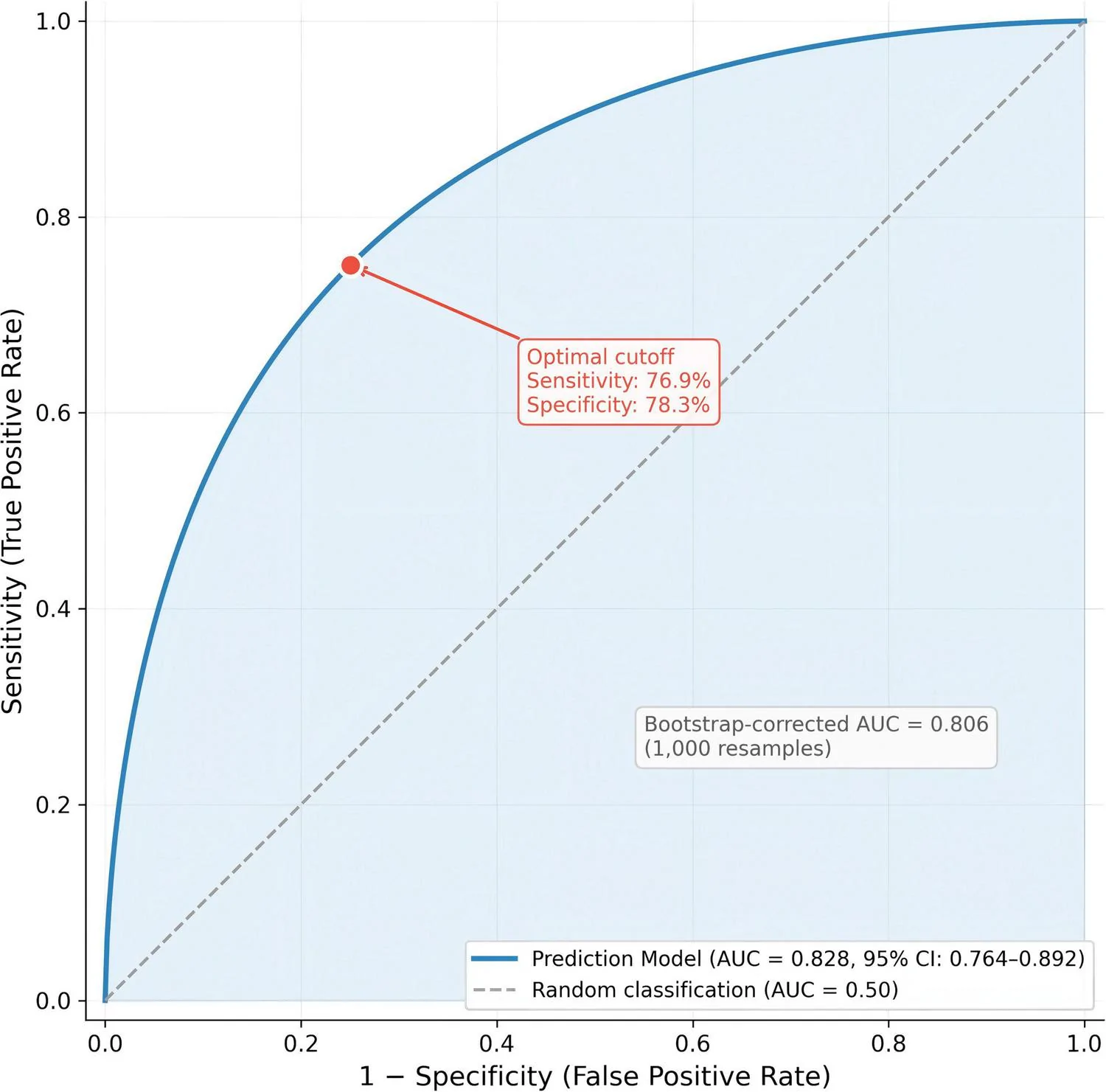
Pre-post biomarker changes in treatment responders versus non-responders, organized by the four sub-dimensions of the brain-gut-immune axis. Bars indicate the mean change and error bars indicate the standard error of the mean. Between-group comparisons of change scores used Welch t-tests for normally distributed markers and Mann-Whitney U tests for skewed markers, with Benjamini-Hochberg false discovery rate correction across the nine biomarkers. Responders showed significantly greater biomarker modulation than non-responders for all markers except 5-HT. ns, not significant; **P < 0.01; ***P < 0.001. β-EP, β-endorphin; LPS, lipopolysaccharide; 5-HT, 5-hydroxytryptamine; IL-6, interleukin-6; TNF-α, tumor necrosis factor-α; IL-10, interleukin-10; hs-CRP, high-sensitivity C-reactive protein.

To examine whether biomarker modulation tracked clinical response, post-treatment biomarker changes were compared between responders and non-responders (Table 6 and Figure 5). Responders showed significantly greater reductions in cortisol, LPS, D-lactate, IL-6, TNF-α, and hs-CRP, and significantly greater increases in β-EP and IL-10, than non-responders (all FDR-adjusted *P* < 0.05), whereas the change in 5-HT did not differ between groups (*P* = 0.302). Within non-responders, the changes in IL-6, IL-10, and 5-HT did not reach statistical significance, in contrast to the uniformly significant changes observed in responders. This graded concordance between symptomatic relief and biomarker modulation strengthens the biological plausibility of the proposed mechanism and indicates that the biomarker shifts were not uniform artifacts of study participation. These between-group differences were robust to the responder definition: when treatment response was instead defined as a ≥ 30% VAS reduction (155 responders, 83.3%), responders again showed significantly greater modulation of β-EP, IL-6, LPS, and hs-CRP than non-responders (all *P* < 0.01).

**TABLE 6:** Pre–post biomarker changes in treatment responders (*n* = 117) versus non-responders (*n* = 69).

Biomarker	Group	Baseline	Post-treatment	Within-group P	Δ (mean)	Between-group P	FDR P
β-EP (pg/mL)	Responder	152.7 ± 41.2	213.4 ± 53.5	< 0.001	+60.74	< 0.001	<0.001
	Non-resp.	158.3 ± 43.5	176.3 ± 59.2	< 0.001	+17.96		
Cortisol (μg/dL)	Responder	18.8 ± 4.7	14.1 ± 6.3	< 0.001	−4.77	< 0.001	<0.001
	Non-resp.	18.0 ± 4.5	15.4 ± 5.6	< 0.001	−2.57		
LPS (EU/mL)	Responder	36.3 (24.6–53.0)	27.9 (14.2–41.6)	< 0.001	−10.00	< 0.001	0.002
	Non-resp.	20.1 (12.8–33.6)	15.7 (4.6–32.0)	< 0.001	−4.87		
D-lactate (μg/mL)	Responder	16.6 (11.9–26.3)	12.1 (5.8–20.4)	< 0.001	−5.46	0.009	0.010
	Non-resp.	15.8 (10.6–23.2)	13.4 (7.0–21.7)	< 0.001	−3.02		
5-HT (ng/mL)	Responder	122.9 ± 38.6	130.7 ± 54.5	0.023	+7.86	0.302	0.302
	Non-resp.	118.7 ± 36.7	121.3 ± 46.4	0.486	+2.62		
IL-6 (pg/mL)	Responder	8.9 (6.3–15.8)	6.2 (1.6–12.3)	< 0.001	−3.92	< 0.001	<0.001
	Non-resp.	7.9 (4.4–11.7)	7.5 (3.5–11.9)	0.095	−0.76		
TNF-α (pg/mL)	Responder	11.9 (7.2–17.4)	8.0 (1.8–13.4)	< 0.001	−3.82	0.003	0.004
	Non-resp.	10.9 (7.4–15.8)	8.2 (4.3–16.8)	0.008	−1.51		
IL-10 (pg/mL)	Responder	14.2 ± 5.7	19.3 ± 9.3	< 0.001	+5.03	0.001	0.002
	Non-resp.	15.2 ± 5.7	16.2 ± 9.9	0.295	+1.03		
hs-CRP (mg/L)	Responder	5.2 ± 2.9	3.2 ± 3.0	< 0.001	−2.06	< 0.001	<0.001
	Non-resp.	4.2 ± 2.1	3.7 ± 2.8	0.048	−0.51		

Δ, change score (post-treatment minus baseline). Within-group *P*-values are from paired *t*-tests or Wilcoxon signed-rank tests; between-group *P*-values compare Δ between the two groups using Welch *t*-tests or Mann-Whitney U tests, with FDR P the Benjamini-Hochberg-adjusted between-group P across the nine biomarkers. Treatment response was defined as ≥ 50% VAS reduction. Non-resp., non-responders; biomarker abbreviations as in Table 2.

### 3.9 Univariable logistic regression

Univariable logistic regression analysis screened 22 candidate predictor variables, including demographic, clinical, and baseline biomarker variables (Table 7). Variables achieving *P* < 0.20 and thus entering the multivariable model included: baseline VAS (OR = 1.82, 95% CI: 1.38–2.40, *P* < 0.001), mean heat-sensitization intensity (OR = 4.28, 95% CI: 2.46–7.45, *P* < 0.001), baseline LPS (OR = 1.03, 95% CI: 1.01–1.05, *P* = 0.002), baseline ODI (OR = 1.04, 95% CI: 1.01–1.07, *P* = 0.018), baseline IL-6 (OR = 1.06, 95% CI: 1.01–1.11, *P* = 0.008), baseline hs-CRP (OR = 1.14, 95% CI: 1.02–1.27, *P* = 0.015), and pain duration (OR = 1.01, 95% CI: 0.99–1.02, *P* = 0.182).

**TABLE 7:** Univariable logistic regression for predictors of treatment response (selected variables).

Variable	OR (95% CI)	Wald χ ^2^	*P*-value
Age	1.01 (0.98–1.04)	0.40	0.528
Sex (female)	1.05 (0.58–1.92)	0.03	0.868
Pain duration	1.01 (0.99–1.02)	1.78	0.182
Baseline VAS	1.82 (1.38–2.40)	17.64	< 0.001
Baseline ODI	1.04 (1.01–1.07)	5.58	0.018
Mean HSI score	4.28 (2.46–7.45)	24.32	< 0.001
Baseline β-EP	1.00 (0.99–1.01)	0.52	0.470
Baseline Cortisol	1.04 (0.97–1.11)	1.24	0.266
Baseline LPS	1.03 (1.01–1.05)	9.42	0.002
Baseline 5-HT	1.00 (0.99–1.01)	0.52	0.472
Baseline IL-6	1.06 (1.01–1.11)	7.02	0.008
Baseline hs-CRP	1.14 (1.02–1.27)	5.94	0.015

OR, odds ratio; CI, confidence interval; HSI, heat-sensitization intensity. Variables with *P* < 0.20 entered the multivariable model.

### 3.10 Multivariable logistic regression

The final multivariable logistic regression model retained three independent predictors of treatment response (Table 8): mean heat-sensitization intensity score (adjusted OR = 3.64, 95% CI: 1.98–6.70, *P* < 0.001), baseline VAS (adjusted OR = 1.58, 95% CI: 1.16–2.15, *P* = 0.004), and baseline LPS (adjusted OR = 1.02, 95% CI: 1.00–1.04, *P* = 0.028). The model demonstrated good discriminative performance with an area under the ROC curve of 0.828 (95% CI: 0.764–0.892). The Hosmer-Lemeshow test indicated adequate calibration (χ^2^ = 6.14, *P* = 0.632). Bootstrap internal validation with 1000 resamples yielded an optimism-corrected AUC of 0.806, indicating acceptable internal validity.

**TABLE 8:** Multivariable logistic regression model for treatment response prediction.

Predictor	Adjusted OR (95% CI)	Wald χ ^2^	*P*-value	VIF
Mean HSI score	3.64 (1.98–6.70)	16.28	< 0.001	1.14
Baseline VAS	1.58 (1.16–2.15)	8.34	0.004	1.21
Baseline LPS (EU/mL)	1.02 (1.00–1.04)	4.82	0.028	1.16

Adjusted OR, odds ratio adjusted for the other variables in the model; CI, confidence interval; VIF, variance inflation factor; HSI, heat-sensitization intensity. Model performance: AUC = 0.828 (95% CI: 0.764–0.892); Hosmer-Lemeshow χ^2^ = 6.14, P = 0.632; bootstrap-corrected AUC = 0.806.

### 3.11 Mediation analysis

To explore whether the analgesic effect operated through the brain-gut-immune axis, single-mediator analyses were performed with treatment dose (mean HSI score) as the independent variable, each biomarker change as the mediator, and pain improvement (−ΔVAS) as the outcome, using standardized coefficients and 5,000 bootstrap resamples (Table 9). Significant indirect effects were observed for Δβ-EP (proportion mediated 44.9%; 95% CI 0.095–0.241), ΔIL-6 (37.3%; 0.083–0.193), Δhs-CRP (23.2%; 0.034–0.142), ΔLPS (16.8%; 0.017–0.116), and ΔCortisol (7.9%; 0.001–0.072), with all bootstrap confidence intervals excluding zero. These results are consistent with partial mediation of the analgesic response through endogenous opioid and anti-inflammatory pathways. Given the uncontrolled design and the contemporaneous measurement of mediators and outcome, these estimates are exploratory and hypothesis-generating and do not establish the direction of causation.

**TABLE 9:** Single-mediator analysis of biomarker change mediating the association between treatment dose (mean heat-sensitization intensity) and pain improvement (−ΔVAS).

Mediator (M)	a (X→M)	b (M→Y | X)	Indirect effect (a × b)	95% CI (bootstrap)	Proportion mediated
Δβ-EP	0.412	0.396	0.163	[0.095, 0.241]	44.9%
ΔIL-6	−0.328	−0.413	0.136	[0.083, 0.193]	37.3%
Δhs-CRP	−0.321	−0.262	0.084	[0.034, 0.142]	23.2%
ΔLPS	−0.298	−0.204	0.061	[0.017, 0.116]	16.8%
ΔCortisol	−0.163	−0.175	0.029	[0.001, 0.072]	7.9%

Single-mediator models with treatment dose (mean HSI score) as the independent variable (X), each biomarker change (Δ, post-treatment minus baseline) as the mediator (M), and pain improvement (−ΔVAS) as the outcome (Y), computed with standardized variables. a, X→M path; b, M→Y path adjusted for X; indirect effect = a × b; 95% CIs are bias-corrected bootstrap intervals (5,000 resamples); proportion mediated = indirect effect/total effect. Only biomarkers with a significant indirect effect (bootstrap 95% CI excluding zero) are shown; these analyses are exploratory and do not establish causation. HSI, heat-sensitization intensity; other abbreviations as in Table 2.

### 3.12 ROC curve analysis

**FIGURE 6:**
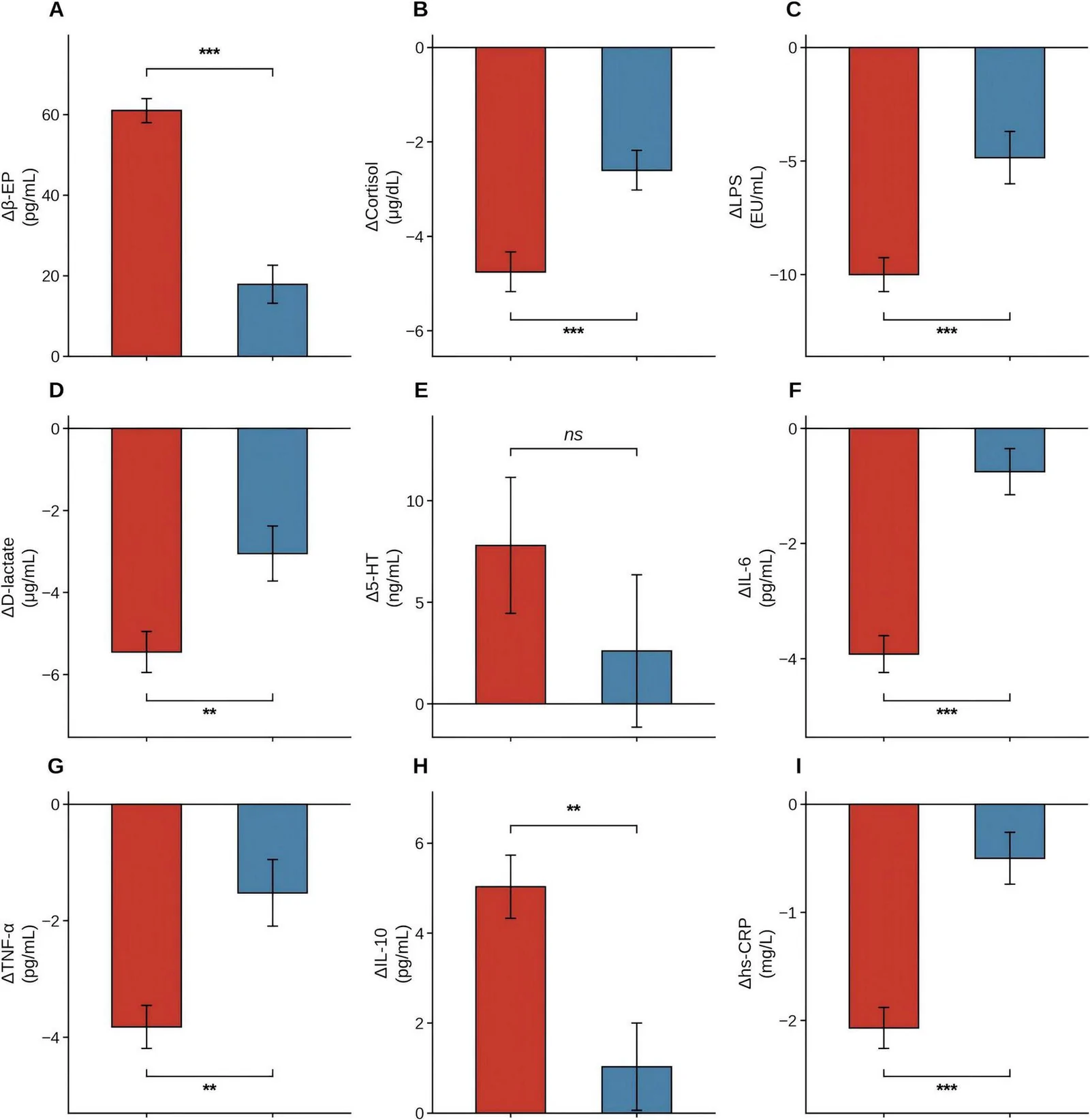
ROC curve for the multivariable logistic regression model predicting treatment response. Predictors retained in the final model were mean heat-sensitization intensity score, baseline VAS, and baseline serum LPS. The area under the curve (AUC) was 0.828 (95% CI: 0.764–0.892); the bootstrap-corrected AUC was 0.806. The dashed diagonal line represents random classification (AUC = 0.50). At the Youden index optimal cutoff, sensitivity = 76.9% and specificity = 78.3%.

The receiver operating characteristic curve for the multivariable prediction model is presented in Figure 6. The AUC of 0.828 indicates good discriminative ability. Using the Youden index to determine the optimal cutoff, the model achieved a sensitivity of 76.9% and a specificity of 78.3% for predicting treatment response.

### 3.13 Safety assessment

The safety profile of HSM is summarized in Table 10. A total of 28 adverse events were reported in 24 patients (12.9%). The most common was mild local erythema at the moxibustion site (*n* = 14, 7.5%), followed by minor blister formation (*n* = 7, 3.8%), transient dizziness during treatment (*n* = 4, 2.2%), and local itching (*n* = 3, 1.6%). All adverse events were mild, self-limiting, and resolved within 1–3 days without intervention. No serious adverse events, burns requiring medical treatment, or treatment discontinuations due to adverse events were reported.

**TABLE 10:** Summary of adverse events during the 4-week HSM treatment (*N* = 186).

Adverse Event	n (%)	Severity	Outcome
Local erythema	14 (7.5%)	Mild	Self-resolved (1–2 days)
Minor blister	7 (3.8%)	Mild	Self-resolved (2–3 days)
Transient dizziness	4 (2.2%)	Mild	Self-resolved (same day)
Local itching	3 (1.6%)	Mild	Self-resolved (1 day)
Total events/patients	28/24 (12.9%)	–	–

HSM, heat-sensitive moxibustion. All adverse events were mild and self-limiting; no serious adverse events occurred.

## 4 Discussion

### 4.1 Clinical efficacy summary

In this exploratory study, a 4-week course of HSM was associated with clinically meaningful reductions in pain and functional disability in patients with CNLBP. The effect sizes were well above the minimal clinically important difference (MCID) of 1.5–2.0 points commonly accepted for chronic pain interventions (Salaffi et al., 2004). The treatment response rate of 62.9%, defined as a VAS reduction of at least 50%, is on a par with, and in some cases above, the response rates of 40–60% reported in earlier acupuncture trials for CNLBP (Vickers et al., 2018). The concurrent improvements in SDS and SAS scores also suggest that the analgesic action of HSM is not limited to peripheral nociceptive modulation but reaches into central affective pain processing as well (Bushnell et al., 2013). The size and consistency of change across several outcome domains, together with biologically meaningful shifts in the brain-gut-immune biomarker panel, are consistent with a treatment-associated effect but, given the uncontrolled single-arm design, cannot establish causal efficacy; they nonetheless set the stage for the mechanistic discussion that follows.

### 4.2 Immune-inflammatory dimension: anti-inflammatory rebalancing

The simultaneous drop in pro-inflammatory cytokines (IL-6, TNF-α, and hs-CRP) and rise in the anti-inflammatory cytokine IL-10 point to a move away from the chronic low-grade inflammatory state believed to drive and sustain CNLBP (Klyne et al., 2017). At the mechanistic level, IL-6 and TNF-α sensitize peripheral nociceptors, promote activation of spinal microglia and astrocytes, and trigger NF-κB-mediated transcription of nociceptive mediators that maintain central sensitization and pain chronicity (Ji et al., 2016). One likely upstream driver of this anti-inflammatory shift is vagally mediated activation of the cholinergic anti-inflammatory pathway (CAIP). Somatosensory and thermal afferent input from heat-sensitized acupoints is thought to engage vagal afferent fibers that project to the nucleus tractus solitarius. Reflex efferent vagal signaling then releases acetylcholine, which binds α7 nicotinic acetylcholine receptors on tissue macrophages and suppresses NF-κB-dependent cytokine production (Tracey, 2002). The matching rise in IL-10, an anti-inflammatory cytokine that inhibits IL-6 and TNF-α synthesis through JAK1-STAT3 signaling and SOCS3 induction, suggests that an active immunomodulatory program is in play rather than passive resolution of inflammation (Saraiva and O’Garra, 2010). The observed correlation between ΔIL-6 and ΔVAS in this cohort is consistent with a relationship between peripheral immune rebalancing and analgesic response, in keeping with the broader concept of cytokine-driven central sensitization in chronic musculoskeletal pain (Ji et al., 2016). This anti-inflammatory pattern is consistent with a systematic review and meta-analysis of 30 randomized controlled trials showing that moxibustion reduces circulating pro-inflammatory cytokines, including IL-6 and TNF-α, in patients with low back pain (Zhao et al., 2023).

### 4.3 Neuroendocrine dimension: endogenous opioid activation and HPA axis normalization

The post-HSM rise in serum β-EP gives correlative backing to the idea that activation of the endogenous opioid system is a core analgesic mechanism. β-EP is the main endogenous ligand at μ-opioid receptors. It is released from the hypothalamic arcuate nucleus and pituitary corticotropes in response to noxious and somatosensory afferent input, and contributes to descending pain inhibition through projections to the periaqueductal gray (PAG) and the rostral ventromedial medulla (Fields, 2004). The strong inverse correlation between Δβ-EP and ΔVAS in this cohort fits with classical evidence that analgesia produced by acupuncture and moxibustion is at least partly reversed by the opioid antagonist naloxone (Pomeranz and Chiu, 1976). It should be noted, however, that this naloxone-reversibility evidence derives largely from acupuncture paradigms, and direct evidence for endogenous opioid involvement specific to moxibustion remains limited; the present β-EP findings are therefore correlative and should be interpreted with this caveat.

The parallel drop in serum cortisol suggests that the hyperactivity of the HPA axis, a well-described feature of chronic pain, has been partly corrected. Long-term glucocorticoid exposure has several harmful effects relevant to CNLBP. It strengthens spinal nociceptive transmission by glucocorticoid receptor-mediated potentiation of glutamatergic signaling, it suppresses hippocampal neurogenesis thought to take part in pain memory consolidation (Mutso et al., 2012), and it disrupts intestinal tight-junction architecture by upregulating claudin-2 while downregulating occludin and ZO-1 (De Punder and Pruimboom, 2015). Normalization of cortisol may therefore act as an upstream hinge that links the neuroendocrine, intestinal barrier, and immune dimensions of the brain-gut-immune axis, in which a single neuroendocrine correction yields coordinated downstream benefits across several compartments of the system.

### 4.4 Intestinal barrier dimension: restoration of gut barrier integrity

The reductions in serum LPS and D-lactate after HSM are in line with improved intestinal barrier function. These markers were quantified by the LAL chromogenic and colorimetric enzymatic protocols described in the Methods, and heat inactivation together with spike-and-recovery validation were used to limit the known serum protein interference of the LAL assay (Harm et al., 2024). Within the brain-gut-immune framework, HPA hyperactivity driven by chronic pain disrupts paracellular tight junctions and permits translocation of gram-negative bacterial endotoxin and microbial fermentation products into the systemic circulation, a state known as metabolic endotoxemia (Mohammad and Thiemermann, 2020). Circulating LPS engages the TLR4-MD2 complex on innate immune cells, activates MyD88-dependent NF-κB signaling, and amplifies production of IL-6, TNF-α, and other pro-inflammatory mediators that sensitize nociceptive pathways and keep pain going (Mohammad and Thiemermann, 2020). Consistent with an immunomodulatory action of moxibustion on this pathway, preclinical work has shown that moxibustion activates macrophage autophagy and enhances host defense against bacterial (endotoxin-bearing) challenge (Li et al., 2014).

The gut-disc axis framework recently put forward for spinal pathology (Morimoto et al., 2023) is particularly relevant to CNLBP. It proposes that translocated LPS may directly engage innate immune receptors on intervertebral disc cells, thus accelerating disc degeneration and adding to nociceptive input from the lumbar segment. The finding that ΔLPS correlated with both ΔIL-6 and ΔTNF-α gives correlative support to the leaky gut driven systemic inflammation axis in a non-visceral musculoskeletal pain population, and the strong correlation between ΔD-lactate and ΔLPS confirms the convergent validity of these two complementary barrier markers (Wang et al., 2025). The retention of baseline LPS as an independent predictor in the multivariable model further suggests that intestinal permeability may shape treatment responsiveness. This finding deserves independent replication using direct measures of barrier function such as zonulin or intestinal fatty acid-binding protein, alongside fecal microbiome profiling (Vanuytsel et al., 2021).

### 4.5 Gut-brain serotonergic signaling dimension

Serum 5-HT showed a numerical increase that did not survive FDR correction, with a small effect size and substantial variation between individuals. This pattern should be read with caution given the inherent limits of serum 5-HT as a surrogate for gut-brain serotonergic tone, as already noted in the Methods. Platelets sequester roughly 95% of circulating 5-HT through SERT and release it during clot formation, so serum 5-HT measurements largely reflect platelet serotonin content rather than free plasma 5-HT or direct enterochromaffin cell output (Mercado and Kilic, 2010). Variation in platelet count, platelet turnover, SERT expression, and clotting kinetics may therefore confound the interpretation of serum 5-HT changes, independent of true gut-derived serotonergic activity.

Despite this limit, the weak but statistically significant inverse correlation between Δ5-HT and ΔSDS, together with a trend-level association with ΔSAS, is biologically coherent. About 90% of total body serotonin is synthesized in intestinal enterochromaffin cells, and gut-derived 5-HT can influence central nervous system function indirectly through vagal afferent activation and modulation of central serotonergic circuits that project to limbic structures involved in mood regulation (Yano et al., 2015). The absence of a meaningful correlation between Δ5-HT and either ΔVAS or ΔODI suggests that the serotonergic modulation brought about by HSM, when it occurs, may engage the affective-motivational rather than the sensory-discriminative dimension of pain (Price, 2000). Future studies that use platelet-poor plasma 5-HT, urinary 5-hydroxyindoleacetic acid, or stool serotonergic metabolites would give a more specific readout of gut-derived serotonergic signaling.

### 4.6 Integrated brain-gut-immune axis modulation model

Combining the four sub-dimensions, an integrated mechanistic model can be proposed for HSM analgesia, made up of four parallel but interconnected pathways. Thermal stimulation of heat-sensitized acupoints is thought to engage cutaneous and subcutaneous TRPV1/TRPV2 channels on Aδ and C fiber sensory endings (Jiang et al., 2016). The resulting afferent input is proposed to ascend through somatosensory and vagal pathways to the central nervous system, where it may initiate four downstream cascades; because the study lacked a control arm, it is not possible to determine which component of the intervention (for example, the specific thermal-sensory stimulus versus the opportunity for approximately 1 h of daily relaxation and therapeutic contact) accounted for the observed changes, and the following pathway model is therefore presented as a mechanistic hypothesis rather than a demonstrated causal chain. In the neuroendocrine pathway, brainstem and hypothalamic relays trigger β-EP release from the arcuate nucleus, which activates descending μ-opioidergic inhibition through projections from PAG to the rostral ventromedial medulla (Fields, 2004), and parasympathetic recruitment at the same time dampens HPA axis output and lowers cortisol. In the intestinal barrier pathway, cortisol normalization allows tight-junction integrity to be restored, which reduces translocation of LPS and D-lactate (De Punder and Pruimboom, 2015). In the gut-brain serotonergic pathway, normalization of the intestinal environment may modulate 5-HT secretion by enterochromaffin cells, although the heterogeneous response seen in this cohort suggests that this pathway is weaker or more conditional than the others. In the immune-inflammatory pathway, vagally mediated CAIP activation, together with reduced LPS-driven TLR4/NF-κB signaling, produces a coordinated shift toward an anti-inflammatory cytokine profile (Tracey, 2002).

Importantly, these four pathways are unlikely to be independent. We hypothesize that they may form a mutually reinforcing loop in which HPA normalization restores barrier integrity, which then curtails endotoxin-driven inflammation, which in turn reduces nociceptive drive and central stress responses, and so further stabilizes the HPA axis; however, the present cross-sectional biomarker design cannot establish such causal feedback, and this integrated model should be regarded as a hypothesis to be tested rather than an established mechanism. Partial and correlative support for the individual path relationships is nonetheless provided by the observed associations between ΔLPS and both ΔIL-6 and ΔTNF-α and by the mediation analysis reported above (Table 9), while acknowledging that these analyses are not causal. Recent causal evidence that fecal microbiota transplantation from fibromyalgia patients is enough to induce mechanical and thermal hypersensitivity in germ-free mice (Cai et al., 2025) indicates that the gut-immune compartment can act as a primary driver of chronic non-visceral pain, which gives biological weight to the inclusion of baseline LPS as an independent predictor of response. Taken together, these observations identify the gut barrier and immune node as a potentially key therapeutic target within the integrative brain-gut-immune model.

### 4.7 Predictors of treatment response: clinical implications

Three independent predictors, namely mean heat-sensitization intensity, baseline VAS, and baseline LPS, were retained in the multivariable model, with good discriminative performance (AUC = 0.828; bootstrap-corrected AUC = 0.806). The events-per-variable ratio of 39 is well above the conventional threshold of 10, which supports the stability of the parameter estimates (Peduzzi et al., 1996). Heat-sensitization intensity came out as the strongest predictor, a finding with both theoretical and practical importance. Acupoint heat sensitization is thought to reflect a functionally activated state of the acupoint, in which afferent neuromodulatory capacity is heightened, possibly through neurogenic inflammation and local sensitization of primary afferents (Tan et al., 2019). Supporting neurophysiological evidence comes from EEG source analyses that show distinct cortical activation patterns during heat-sensitization responses, which suggests a measurable central correlate of the clinical phenomenon (Wang et al., 2015). As far as is known, this is the first study to formally model heat-sensitization intensity as a continuous predictor in a multivariable logistic regression framework, addressing a methodological gap in the quantitative modeling of heat-sensitization phenomena. This predictor is conceptually similar to the temporal summation of pain used by Baeumler and colleagues to predict acupuncture response (Baeumler et al., 2019), in that both index central neuroplastic capacity rather than baseline pain severity as such.

The inclusion of baseline LPS as a predictor is novel and clinically informative. Patients with more baseline barrier disruption may benefit more from HSM, because the gut barrier and immune restoration pathway has more room to be corrected in this subgroup. The same principle has been seen across other interventions, where larger baseline pathophysiological deviation predicts a larger treatment effect (Kraemer et al., 2002). If this finding is replicated, baseline LPS could serve as a stratification biomarker that enables enrichment designs in future trials and more personalized application in clinical practice. The retention of baseline VAS as a third predictor fits with a recurring observation in pain medicine, namely that patients with a higher initial symptom burden have more absolute room for improvement, while heat-sensitization intensity captures the patient’s own responsiveness to the intervention.

### 4.8 Comparison with prior studies

The present study extends the existing literature in three directions. In the mechanistic domain, recent work showing that fecal microbiota transplantation from fibromyalgia patients induces nociceptive hypersensitivity in germ-free mice (Cai et al., 2025), together with Mendelian randomization evidence that links specific gut taxa and metabolites to low back pain (Ma, 2025; Su et al., 2023), establishes the gut-brain-pain axis as a causal contributor to chronic non-visceral pain. The present findings add complementary therapeutic evidence that an external intervention can engage this axis and produce coherent biomarker shifts alongside symptom improvement, although firm causal attribution will require randomized confirmation. In the prediction domain, the AUC of 0.828 reached by this model compares favorably with earlier LASSO models for electroacupuncture response prediction in chronic low back pain (Kong et al., 2020), which possibly reflects the additional value of including a mechanistically grounded biomarker (LPS) alongside clinical and procedural variables. In the conceptual domain, the agreement between heat-sensitization intensity in the present model and the temporal summation predictor identified by Baeumler et al. (2019) strengthens the broader hypothesis that central sensitization-related phenotypes may be a unifying determinant of responsiveness to somatosensory interventions across different modalities.

### 4.9 Limitations

Several limitations should be acknowledged when interpreting the present findings. Although the sample of 186 completers is relatively large for an exploratory moxibustion study, it is still not large enough for reliable subgroup stratification analyses based on baseline 5-HT phenotype or inflammatory phenotype. The 4-week intervention period without later follow-up assessment also makes it impossible to judge the durability of the effect or the chance of symptom relapse. Fecal microbiome sequencing (16S rRNA or shotgun metagenomics) was not carried out, so the analysis relies on serum LPS and D-lactate, which are only indirect surrogate markers of intestinal permeability and cannot characterize gut microbial composition or specific barrier components. The validation of these markers has also come mainly from surgical and gastroenterological cohorts and remains poorly established in musculoskeletal pain populations (Seethaler et al., 2021; Vanuytsel et al., 2021). A further limitation is that the use of a single baseline and post-treatment measurement design cannot resolve the temporal dynamics or dose-response relationships of biomarker change. Finally, the unblinded design exposes subjective outcome measures to expectation bias, and although the biomarker panel provides an objective complement, this limitation cannot be fully removed. Nonetheless, although non-responders reported less than 50% VAS reduction, they still exhibited significant within-group changes in several objective biomarkers, including β-EP, cortisol, and LPS (Table 6), suggesting that measurable biological effects occurred partly independently of subjective symptom report and providing an objective counterweight to expectation bias, even though such bias cannot be excluded entirely. In addition, because the heat-sensitization intensity was scored from the single dominant heat-sensitized point at each session rather than summed across all sensitized regions, a cumulative multi-region index might capture the sensitization burden more completely and should be considered in future studies. As the gut microbiome was not profiled, any inference regarding microbial mediation of the observed LPS and D-lactate changes remains speculative and requires confirmation with 16S rRNA or shotgun metagenomic sequencing.

## 5 Conclusion

In this exploratory single-arm study, a 4-week course of HSM was followed by clinically meaningful improvements in pain, disability, and psychological outcomes in patients with CNLBP, accompanied by coordinated changes across nine serum biomarkers spanning the neuroendocrine, intestinal barrier, gut-brain serotonergic signaling, and immune-inflammatory dimensions of the brain-gut-immune axis. Baseline heat-sensitization intensity, pain severity, and intestinal permeability emerged as independent predictors of treatment response. As an uncontrolled single-arm study, these findings establish association and biological plausibility but cannot demonstrate the causal efficacy or specificity of HSM; they provide a mechanistic framework that warrants confirmation in a randomized controlled trial with a sham-moxibustion comparator.

## Data Availability

The raw data supporting the conclusions of this article will be made available by the authors, without undue reservation.
